# Revisiting glucose uptake and metabolism in schistosomes: new molecular insights for improved schistosomiasis therapies

**DOI:** 10.3389/fgene.2014.00176

**Published:** 2014-06-11

**Authors:** Hong You, Rachel J. Stephenson, Geoffrey N. Gobert, Donald P. McManus

**Affiliations:** ^1^Molecular Parasitology Laboratory, Infectious Diseases Division, QIMR Berghofer Medical Research InstituteBrisbane, QLD, Australia; ^2^Faculty of Science, School of Chemistry and Molecular Biosciences, The University of QueenslandBrisbane, QLD, Australia

**Keywords:** schistosome, *Schistosoma*, glucose uptake and metabolism, insulin signaling pathway, glycolysis signaling pathway

## Abstract

A better understanding of the molecular mechanisms required for schistosomes to take up glucose, the major nutritional source exploited by these blood flukes from their mammalian hosts and the subsequent metabolism required to fuel growth and fecundity, can provide new avenues for developing novel interventions for the control of schistosomiasis. This aspect of parasitism is particularly important to paired adult schistosomes, due to their considerable requirements for the energy needed to produce the extensive numbers of eggs laid daily by the female worm. This review describes recent advances in characterizing glucose metabolism in adult schistosomes. Potential intervention targets are discussed within the insulin signaling and glycolysis pathways, both of which play critical roles in the carbohydrate and energy requirements of schistosomes.

## Introduction

Schistosomiasis remains one of the most devastating tropical parasitic diseases (Colley et al., 2014), with an estimated 200 million people infected (85% live in Africa), and about 700 million people at risk in 74 countries (Nour, 2010). This is despite the availability of a highly effective drug [praziquantel (PZQ)] and extensive ongoing control programs involving mainly mass drug administration. Important shortcomings of PZQ include its relative inactivity against migratory juveniles and developing worms (Gonnert and Andrews, 1977) and its inability to prevent reinfection. Schistosomes have a complex life cycle, with discrete stages perfectly adapted to their differing host and free-living environments, to promote survival and transmission. The mammalian endoparasitic life cycle begins with the penetration of cercariae, released from freshwater snails, though host skin; then these larvae develop into schistosomula which move to the lungs and pass down the mesenteric vasculature (*S. mansoni, S. japonicum*) or urinary bladder venous plexus (*S. haematobium*) where host signals stimulate further development of the juvenile males and females. Pairing and sexual maturation of the adult worms results in extensive egg production. It is the schistosome eggs that are responsible for both the severe pathology associated with schistosomiasis, due to granuloma formation around the ova trapped in tissues, and transmission. The latter is dependent upon the eggs being released from the definitive mammalian host into water and their hatching to release miracidia which penetrate the appropriate freshwater snail intermediate host. Within the haemocoel of the snail, the miracidia form sporocysts, in which further asexual propagation releases larval cercariae. Such processes of multiplication and proliferation are highly energy consuming and schistosomes are entirely reliant on their hosts for the essential nutrients they require for development, reproduction and metabolism.

It is anticipated that better strategies, including vaccines, for schistosomiasis control will rely on an improved understanding of how schistosomes utilize host nutrients, neuro-endocrine hormones and signaling for their survival, development and maturation. Comprehensive deciphering of the available schistosome genomes, transcriptomes, and proteomes is becoming increasingly important for understanding the highly adapted relationship between parasite and hosts on the path to identifying novel drug or vaccine targets (Berriman et al., 2009; *Schistosoma japonicum* Genome Sequencing and Functional Analysis Consortium, 2009; Young et al., 2012). Resident in the mammalian host bloodstream, schistosomes import essential nutrients across the body surface and through the gut and it is striking that the adult worms consume their dry weight of glucose from the host every 5 h (Bueding, 1950) in order to survive. Here, we focus on the molecular features associated with glucose uptake and metabolism in schistosomes during the intra-mammalian stages of development. We also discuss the essential role these processes play in worm development, growth and maturation, and the potential for the associated molecular components might have as novel vaccine or drug targets.

## Cloning and characterization of schistosome glucose transporter proteins

It is recognized that glucose is the common currency of cellular metabolism and all cells import glucose across their hydrophobic surface membranes using glucose transporter proteins (GTPs) (Lienhard et al., 1992). Four glucose transporters (SGTP1, 2, 3, and 4) have been identified in *S. mansoni* (Skelly et al., 1994; Krautz-Peterson et al., 2010), of which SGTP1 and SGTP4 display glucose transport activity, and are markedly inhibited by cytochalasin B in *Xenopus* uptake assays (Skelly et al., 1994). *SGTP1* is present in a number of life stages (eggs, sporocysts, cercariae, schistosomula and adult female and male worms) while *SGTP4* has been detected only in the intra-mammalian forms, where it appears after the transformation of the cercariae into schistosomula and the appearance of the double membrane of the adult worm tegument (Skelly and Shoemaker, 1996). Both SGTP1 and SGTP4 are localized to the tegument of adult worms and schistosomula (Skelly and Shoemaker, 1996). SGTP4 seems to be localized uniquely to the apical membranes of the tegument, while SGTP1 is located on the tegumental basal membrane and within the worm body, particularly in muscle. This localization of the two SGTPs in the schistosome tegument implies that the host-interactive SGTP4 protein facilitates the import of glucose from the host bloodstream into the tegument while SGTP1 serves to move free glucose from the surface into the interstitial fluids providing a nutrient source for the internal tissues (Zhong et al., 1995). Probing of cryosections of adult *S. japonicum* with anti-SGTP1 and SGTP4 antibodies showed the same localization for SGTP1 and SGTP4 in the tegument of *S. japonicum* and *S. mansoni* suggesting sequence homology between the SGTPs of the two species (Jiang et al., 1999). Sequencing of the *S. japonicum* genome has indicated this is the case for *SjGTP1* although only partial sequence is available for *SjGTP4* (http://chgc.sh.cn/japonicum/) (Supplementary Table 1). Full length sequences for *GTP1* and *GTP4* have also been identified in *S. haematobium* (Young et al., 2012) (Supplementary Table 1). RNAi studies have shown that *SGTP1*- or *SGTP4*-suppressed schistosomula and adult worms of *S. mansoni* have an impaired ability to transfer glucose (Krautz-Peterson et al., 2010). Worms having both *SGTP1* and *SGTP4* suppressed showed a further decreased capacity to take up glucose compared with worms having only a single *SGTP* gene knocked down. Significantly fewer parasites having *SGTP*-knocked down survived after prolonged culture in glucose-depleted medium compared with controls. Further, *SGTP*-knockdown parasites exhibited less viability *in vivo* after infection of mice. Taken as a whole, these studies emphasized the important roles of SGTP1 and SGTP4 in the transfer into schistosomes of exogenous glucose, which is the major energy source for parasite survival and development in the mammalian host.

In addition, SGTP2 and SGTP3 are also suspected to be associated with glucose uptake in schistosomes. SGTP2 expression was shown to be limited to the female reproductive tract (Skelly et al., 1998) but the protein did not appear to be functional in glucose transport and it was speculated that a deletion mutation in SGTP2 occurred in its recent evolutionary history resulting in its loss of function (Skelly et al., 1994). *In silico* annotation has indicated that SGTP3 (Smp_127200) is the most recently identified component considered as a potential glucose transporter in *S. mansoni* (Krautz-Peterson et al., 2010) but further functional investigation is required to confirm this observation.

## Signal transduction regulates schistosome glucose import

When considering GTPs in mammalian cells, it is impossible to ignore the important role of insulin in the dual regulation of increasing glucose uptake and glycogen synthesis (glycogenesis). The *Caenorhabditis elegans* genome encodes a single insulin/IGF-1-like receptor (*daf-2*) and 40 members of insulin-like peptides (*ILPs*) (Li et al., 2003). Recently, it has been confirmed that in *C. elegans*, daf-2 modulates glucose transport via the insulin signaling pathway, in similar manner to that found in mammalian cells (Beall and Pearce, 2002). Whether insulin regulates glucose uptake in schistosomes by a similar mechanism to that observed in *C. elegans* remains to be determined. Schistosomes are unable to synthesize insulin (2009) although insulin-degrading proteases (inferred from annotation) have been identified in *S. japonicum* (Sjp_0009920, http://chgc.sh.cn/japonicum/) and *S. mansoni* (Smp_128100, http://www.genedb.org/Homepage/Smansoni) which may have the same ability to degrade the B chain of host insulin as mammalian cells (Affholter et al., 1988). Microarray analysis demonstrated that adult worms of *S. japonicum* depend on host insulin for growth and fecundity (You et al., 2009). Insulin stimulates glucose uptake, improves the viability of schistosome larvae *in vitro* (Vicogne et al., 2004) and promotes the metabolism and development of adult worms (Saule et al., 2005). These findings contrast with earlier reports which suggested that insulin does not affect glucose consumption by schistosomes*in vitro* (Clemens and Basch, 1989), and that most glucose is imported by adult worms via carrier-mediated diffusion (Skelly et al., 1998). The more recent study by Ahier et al. (2008) has stressed the importance of insulin in modulating glucose uptake in *S. mansoni*. Accordingly, two types of insulin receptors, belonging to the large class of receptor tyrosine kinases (RTKs), have been isolated from *S. mansoni* (SmIR1 and 2) (Khayath et al., 2007) and *S. japonicum* (SjIR1 and 2) (You et al., 2010), both of which are able to bind human insulin. Transcription levels of the *SjIR*s were shown to be up-regulated in mammalian stage parasites (adult worms and schistosomula), further underpinning their involvement in the host-schistosome interaction (You et al., 2010). The SjIRs are also highly expressed in vitelline gland tissue suggesting an important role in supplying nutrients and shell precursors for egg production, the major function of the vitellaria (You et al., 2010). Whereas immunolocalization analysis showed that SmIR1 and SjIR1 are located at the basal membrane of the tegument and in muscles of adult worms (Khayath et al., 2007; You et al., 2010), and have the same location as SGTP1 and SGTP4 (Skelly et al., 1994). While SmIR2 and SjIR2 are, instead, expressed in parenchymal cells of adult males and vitelline cells of females, indicating the two receptor types could have different functions (Khayath et al., 2007). On scrutiny of the recently published *S. haematobium* genomic sequence (Young et al., 2012) by protein BLAST, we identified only *IR2*, although it is likely that *IR1* is also present. Further information on insulin receptors in the three main clinically relevant schistosome species is presented in Supplementary Table 1.

Notably, in a murine vaccine/challenge model of *S.japonicum*, immunization with the L1 subdomain (which may contain the insulin binding sites) of the SjIR fusion proteins expressed in *E. coli*, resulted in highly significant reductions in fecal eggs (56–67%), stunting of adult worms (12–42%), a reduction in hepatic granuloma density (55%) and a reduction in the numbers of mature intestinal eggs (75%) (You et al., 2012). The depression in the development of mature eggs following SjIR vaccination supports earlier observations that a low level of insulin in host blood might hamper egg passage through the intestinal tissue (Hulstijn et al., 2001).

The development of a safe, stable and effective vaccine based on the ligand domains of SjIR1 and 2 using peptides derived from their primary sequences, which are highly antigenic with the ability to bind human insulin, may be feasible due to their low homology to human IR (HIR). Additionally, the HIR contains two receptor binding sites, which are the L1 domain and the first and second type fibronectin III repeats of the insulin receptor (Whittaker et al., 2008). The identification and characterization of the fibronectin domains in the SjIRs, which have considerably lower homology to HIR—but may be important in changing the conformation of the kinase domain and through inducing signaling transduction after binding with insulin—may act as additional targets for blocking or interrupting the binding between the SjIRs and host insulin.

**Figure 1:**
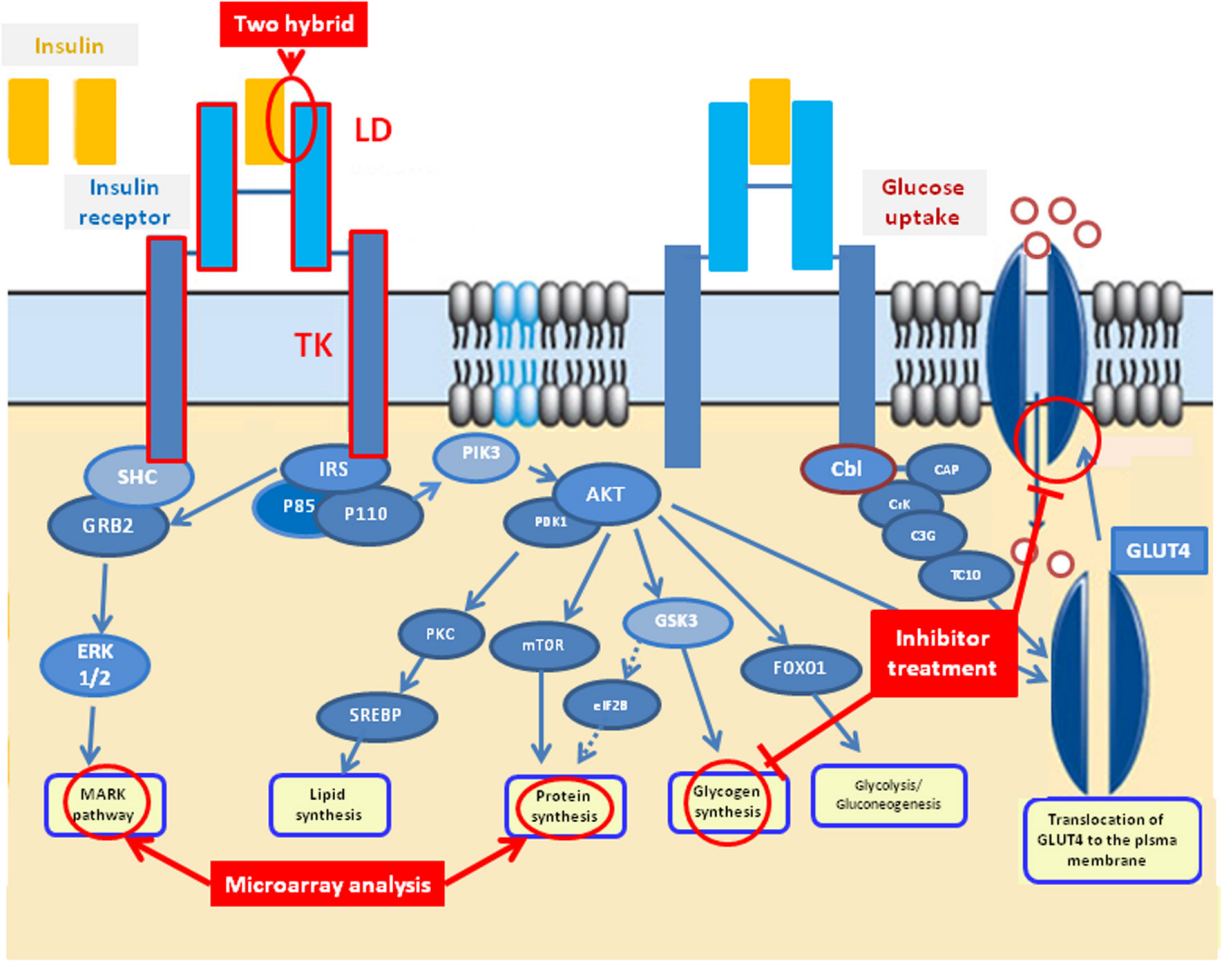
**Predicted insulin signaling pathway in schistosomes based on the characterization of schistosome insulin receptors, the published *S. japonicum* KEGG insulin pathway (www.chgc.sh.cn/japonicum/sjpathway) and the human insulin pathway (http://www.google.com/imgres?imgurl=&imgrefurl=http%3A%2F%2Fwww.abcam.com%2Findex.html%3Fpageconfig%3Dresource%26rid%3D10602&h=0&w=0&tbnid=6ZKuhlAsT2VZXM&zoom=1&tbnh=184&tbnw=274&docid=ETqKf7RmabxHnM&tbm=isch&ei=IU2JU6fSDKmysQSx34BQ&ved=0CAIQsCUoAA)**.

Insulin signaling is an important metabolic pathway in regulating glucose uptake and glycogen synthesis in mammals. Interrogation of the KEGG pathways assigned to metabolic processes indicates a complete insulin signaling pathway is present in *S. japonicum* (www.chgc.sh.cn/japonicum/sjpathway), comprising 43 genes with high homology with other species. Based on published genome data, the 43 genes involved in the insulin pathway have also been identified in *S. mansoni* (Berriman et al., 2009) and *S. haematobium* (Young et al., 2012) (Supplementary Table 2), further supporting the existence of a complete insulin signaling pathway in schistosomes. The available information strongly supports a similar role for schistosome IRs in downstream signal transduction for regulating glucose uptake as occurs in mammalian cells (Figure 1). Both *S. japonicum* and *S. mansoni* IRs have conserved α2β2 structures and structure modeling analysis showed the conserved structure between the SjIRs and HIR, indicating a common predicted binding interaction which occurs in the ligand domain, inducing the same downstream signal transduction in the tyrosine kinase domain (You et al., 2010). Several approaches including two-hybrid analysis, microarray analysis and inhibitor studies *in vitro* (Figure 1) have been used to further support the hypothesis that glucose uptake in adult schistosome worms depends on phosphorylation processes that could be modulated by insulin pathways.

Tyrphostin AG1024 is a potent inhibitor of the RTK venus kinase receptors of *S. mansoni* (Ahier et al., 2008; Vanderstraete et al., 2013b). The venus kinase receptors, named because they contain an atypical venus flytrap (VFT) motif in their extracellular domain, usually present in G-protein coupled C class receptors; the catalytic domains of SmIR1 and SmIR2 and venus kinase receptors (SmVKR1 and SmVKR2) are similar (Vanderstraete et al., 2013b). Both receptor types are important for key biological processes in *S. mansoni* including metabolism and reproduction (Vanderstraete et al., 2013a) and the IRs may be essential in regulating glucose uptake (You et al., 2010). Tyrphostin AG1024 was shown to simultaneously cause inhibition of the functional activity of SmIRs and SmVKRs leading to the killing of both immature and adult *S. mansoni in vitro*. This drug may prove useful for the future design of anti-kinase compounds for anti-schistosome chemotherapy, and as an alternative drug to PZQ, which has no effect on immature worms (Vanderstraete et al., 2013b). SmVKR1 and SmVKR2 are directly associated with parasite growth and fecundity in schistosomes; found only in invertebrates, they are activated by amino-acids (Vicogne et al., 2003) and have an intracellular kinase domain similar to that of SmIRs. These receptors are highly expressed in larval *S. mansoni* and in ovaries of females, indicating involvement in development and reproduction (Gouignard et al., 2012). It is noteworthy that RTKs are considered as attractive anticancer drug or vaccine targets (Arora and Scholar, 2005; Kuwai et al., 2008).

Based on their important involvement in regulating the proliferation and differentiation of vitelline cells and egg embryogenesis (Loverde et al., 2009), parasite RTKs, including IRs and VKRs, transforming growth factor (TGF-β) receptors, epidermal growth factor (EGF) receptor and tumor necrosis factor-α (TNF-α) receptor, have considerable potential as novel intervention candidates against schistosomes and other helminths parasites of clinical and veterinary importance (You et al., 2011).

## Schistosome genes and gene products involved in glycolysis and glycogenesis

During their complex life cycle, schistosomes alternate between consuming host glucose and stored glycogen to provide their energy requirements. When a cercaria penetrates the definitive host and transforms into a schistosomulum, the parasite switches rapidly from carbon dioxide production via the Krebs cycle to lactate production using glycolysis; lactate remains the main end product of glucose degradation as schistosomes develop in their mammalian hosts (Tielens, 1994). In tandem, schistosomes shift rapidly from the consumption of stored glycogen to a dependence on host glucose during the transformation phase from free-living cercariae to schistosomula (Skelly et al., 1993). In the mammalian stages, schistosomula have low expression of respiratory enzymes but regain their capacity for aerobic glucose metabolism as they develop to adult worms (Skelly and Shoemaker, 1995). Schistosomes express a range of mRNAs at relatively high levels associated with anaerobic and oxidative glucose metabolism during the transformation from cercaria to adult, re-emphasizing the fact that adult worms possess a significant capacity to generate energy through aerobic metabolism (Skelly et al., 1993). Glycogen synthesis has been shown to be indirectly proportional to the amount of glycogen already present in adult worms, which in turn is proportional to the size of the parasite (Tielens et al., 1990). It appears that glycogen is degraded intermittently for specific purposes such as muscle contraction or tegumental membrane repair, with both functions being more prevalent in adult males (Gobert et al., 2003). Interrogation of the KEGG pathways assigned to metabolic processes indicates a complete glycolytic pathway is present in *S. japonicum* (www.chgc.sh.cn/japonicum/sjpathway), comprising 23 genes with high homology with other species (Supplementary Figure 1; Supplementary Table 3). Mining the published genomic data for *S. mansoni* (Berriman et al., 2009) and *S. haematobium* (Young et al., 2012) shows all 23 genes involved in glycolysis also occur in these two species (Supplementary Table 3).

A number of the key enzymes involved in the glycolytic pathway in schistosomes have been characterized; these include enolase (Ramajo-Hernandez et al., 2007; De La Torre-Escudero et al., 2010), triose-phosphate isomerize (TPI) (Yu et al., 2006; Da'dara et al., 2008), glyceraldehyde 3-phosphate dehydrogenase (G3PDH) (Goudot-Crozel et al., 1989; Charrier-Ferrara et al., 1992), phosphofructokinase (PFK) (Mansour et al., 2000), phosphoglycerate kinase (PGK) (Lee et al., 1995), hexokinase and glucose-6-phosphatise (Kuser et al., 2000). TPI, which converts glyceraldehyde-3-phosphate to dehydroxyacetone phosphate, is a lead anti-schistosome vaccine candidate (McManus and Loukas, 2008), generating an immune response that presumably reduces the capacity of the blood fluke to metabolize glucose via glycolysis for energy production. TPI is present in most cells of schistosome worms and has also been localized on the surface membranes of the newly transformed schistosomulum (Harn et al., 1992), the stage in the mammalian host that is the likely target of an anti-schistosome vaccine. TPI can induce protection against *S. japonicum* challenge in mice (27.9% worm burden reduction) (Zhu et al., 2002), pigs (48% worm reduction) (Zhu et al., 2006) and water buffaloes (48–52% worm reduction) (Yu et al., 2006; Da'dara et al., 2008). Enolase is another key glycolytic/gluconeogenic enzyme that is a physiological receptor of plasminogen, a molecule which is essential for the activation of the host fibrinolytic system, probably to prevent blood clot formation on the schistosome worm surface (Ramajo-Hernandez et al., 2007; De La Torre-Escudero et al., 2010). Disappointingly, the protective efficacy of recombinant, functional enolase in murine vaccine/*S. japonicum* challenge experiments is marginal (Waine et al., 1993).

## Other components involved in glucose uptake

Acetylcholinesterase (AChE) has been indicated in the modulation of glucose scavenging from mammalian host blood by schistosomes (Camacho and Agnew, 1995). The glucose uptake is regulated by acetylcholine (ACh) interaction with tegumental acetylcholine receptor (nAChR) and AChE. As reviewed by Lee (1996), the secreted AChE may change host cell permeability, have an anti-coagulant role, influence glycogenesis, and play an important function in acetate and choline metabolism as has been shown in nematodes. External AChE may cause the breakdown of local host glycogen stores or block the conversion of glucose to glycogen—as ACh stimulates glycogen synthesis—either of which could make glucose more available in the local environment (Lee, 1996). The function of AChE is supposed to limit the interaction of ACh with its receptor, because inhibition of AChE leads to an effect that mimics ligand excess (Jones et al., 2002). These influences of ACh on glucose uptake can be inhibited through inhibition in turn of either tegumental AChE or tegumental nAChR (Jones et al., 2002). The mammalian stages of schistosomes have AChE and nAChR on their teguments and both components are concentrated on the surface of the adult male, a major surface site for nutrient uptake for the worm pair. The rate of glucose import *in vitro* by *S. haematobium* and *S. bovis* adult worm pairs was shown to be enhanced by approximately 60% at blood (physiological) concentrations of Ach, although *S. mansoni* did not show a similar response (Camacho and Agnew, 1995). It is noteworthy that AChE inhibition in adult worms results in the depletion of tegumental but not muscle glycogen stores; this is relevant since skeletal muscle acts as the primary site for insulin-stimulated glucose disposal in mammals (Thabet et al., 2008). As this observation could not be attributed to the inhibition of glycogen metabolism directly, disruption of sugar transport may have been the cause (Camacho and Agnew, 1995). *In vitro* studies showed that purified polyclonal antibodies against *S. mansoni* AChE were cytotoxic, causing almost total complement-dependent killing of the parasite (Espinoza et al., 1991; Arnon et al., 1999), suggesting AChE may be a highly suitable candidate as a vaccine target, especially as it is highly conserved across a variety of schistosome species (Espinoza et al., 1991) and anti-*S. mansoni* AChE antibodies do not cross-react with human AChE (Espinoza et al., 1991).

As discussed by Hu et al. (2003), cytokines and hormones modulate the integrated response of mammalian hosts to infection by schistosomes. In addition to its essential involvement in the host immune system, an unexpected role for Interleukin-7 (IL-7) has been found in the development, maturation and survival of schistosome worms in mammalian hosts. Infection of mice deficient in IL-7 expression leads to parasite dwarfism (Saule et al., 2002). In this model, IL-7 shares similar, but not identical, effects with the thyroid hormone thyroxin (T4) (Saule et al., 2003). This posed the question as to whether there is a common mediator to their action, which was hypothesized to be host glucose metabolism. Infection with *S. mansoni* resulted in an early peak in glycaemia immediately followed by a peak of insulinemia. In IL-7 + T4 co-treated infected animals, the peak of insulin was abrogated (Saule et al., 2005). The same study further assessed the consequences of experimentally induced glucose- or insulin-level variations on parasite development. Insulin treatment led to increased worm burden and parasite size, thus mimicking the effect of T4 on schistosome development (Saule et al., 2005). Finally, these treatments were also associated with an alteration in the gene expression of schistosome components involved in glucose import (Saule et al., 2005). Overall, IL-7 and T4 regulate schistosome glucose metabolism through modulations in the circulating levels of host glucose and insulin (Saule et al., 2005).

## Other drugs targeting glucose metabolism in schistosomes

Artemether is a highly effective anti-malarial drug that also has anti-schistosomal properties. The precise molecular target of the drug in schistosomes remains unproven although there is some evidence that artemether binds to SmSERCA, a putative Ca^2+^-ATPase of *Schistosoma* (Lepore et al., 2011). Artemether has also been shown to display apparent effects on the carbohydrate metabolism of schistosomes (Zhai et al., 2000b) and key glycolytic enzymes, such as PFK (Xiao et al., 1998) and enolase (Zhai et al., 2000a), any of which might be a target(s) of the drug. Artemether appears to enhance the metabolism of glycogen in adult schistosomes and the inhibition of lactate dehydrogenase thereby reducing the formation of lactate (Xiao et al., 1999). The artemether-induced glycogen decrease in schistosomes was shown to be associated with the inhibition of glycolysis rather than an interference with glucose import (Xiao et al., 1997). Schistosomes recovered from artemether treated animals retain increased glycogen phosphorylase activity, but decreased glucose uptake, due to their decreased glycogen content compared with worms from untreated animals (Shuhua et al., 2000).

Another antimalarial drug mefloquine has shown promise as an antischistosomal agent killing adult schistosomes as well as schistosomula (Manneck et al., 2012). A single dose of mefloquine administered orally to mice infected with *S. mansoni* or *S. japonicum* led to significant reductions in adults and young developing worm burdens of both schistosome species (Keiser et al., 2009). Mefloquine affinity chromatography of crude extracts of *S. mansoni* schistosomula identified one specific mefloquine-binding protein which was the glycolytic enzyme enolase (Manneck et al., 2012). This study also showed that mefloquine and a specific enolase inhibitor—sodium fluoride inhibited enolase activity in crude extracts of schistosomula, although activity of a recombinant form of enolase was unaffected (Manneck et al., 2012). Using isothermal microcalorimetry, the functional inhibition of mefloquine and three known glycolytic pathway inhibitors in schistosomes (sodium fluoride, 3-bromopyruvate, and menadione) were investigated in *S. mansoni* in the presence or absence of glucose. The result suggested a potential role for mefloquine as an inhibitor of glycolysis in lifecycle stages where other targets such as haem degradation are not pertinent (Manneck et al., 2012). Consideration could be given to determine whether mefloquine treatment would be of value in patients who have mixed malaria and *Schistosoma* infections as such coinfections are not uncommon, particularly in Africa, although a concern would be that this strategy might select for *Plasmodium* parasites resistant to mefloquine. Mefloquine monotherapy for schistosomiasis may prove superior to PZQ alone, since both juvenile and mature schistosome worms would be targeted in an infection. Mefloquine, in combination with PZQ, whether as a full or half dose regimen, can substantially reduce the course of infection, thereby further confirming its potential as an anti-schistosomal drug (Nashwa and Abdel-Fattah, 2011). Both artemether and mefloquine have shown promising anti-schistosomal features against adult and juvenile*S. mansoni* in both T cell-deficient mice and in relatively infected age- and sex-matched immunologically intact control mice (Keiser et al., 2010). Artemether treatment reduced total worm burden ranging between 71.1 and 85.3%, while mefloquine induced reductions of total worm number between 80.4 and 97.8% in athymic and immunocompetent NMRI mice. These results suggest that artemether and mefloquine act T-cell independently and that no synergy with the immune response occurs (Keiser et al., 2010). Notably, mefloquine (Lariam) can produce severe neuropsychiatric and psychiatric side effects, which can cause mental health issues (http://www.fda.gov/downloads/Drugs/DrugSafety/ucm088616.pdf), which would be an important consideration for its use in future as an anti-schistosome therapy.

## Concluding remarks and future perspectives

Glucose metabolism leading to ATP synthesis is critical for the survival of schistosomes. Directly or indirectly interrupting or blocking glucose uptake may represent a realistic strategy for drug or vaccine development as it would likely lead to the starvation of worms and an insufficient supply of energy for growth, pairing, maturation, and fecundity.

However, this may not be a simple undertaking as many different components participate in glucose uptake so the blocking or inhibiting of one gene or gene product may stimulate the schistosome worm to compensate by switching to a sub-pathway or another related pathway, so as to allow the acquisition of glucose. One logical approach may be to design multivalent vaccines or drugs targeting two or more key genes that would depress worm growth, reduce worm burden and fecal egg output, with the simultaneous reduction in hepatic egg-associated disease pathology. This strategy could first be developed and tested in the form of a highly efficacious veterinary-based multivalent transmission blocking vaccine (McManus and Loukas, 2008) for application in animal reservoirs of *S. japonicum* in China and the Philippines, and then extended to target the African schistosomes, *S. mansoni*, and *S. haematobium*, with appropriately designed human clinical trials.

### Conflict of interest statement

The authors declare that the research was conducted in the absence of any commercial or financial relationships that could be construed as a potential conflict of interest.
