# Supplementary material for: Revisiting glucose uptake and metabolism in schistosomes: new molecular insights for improved schistosomiasis therapies
Source: Front Genet. 2014 Jun 11;5:176. doi: 10.3389/fgene.2014.00176 (PMC4052099; doi:10.3389/fgene.2014.00176)
Supplement: Supplementary file 1 [file DataSheet1.ZIP › Supp Mat captions.pdf]

Supplementary documents

Supplementary Figure 1. Predicted glycolytic pathway in schistosomes. The score, E value and identity of the *S. japonicum* genes involved in the glycolytic pathway are described at http://chgc.sh.cn/japonicum/Sjpathway/.

Supplementary Table 1. Homology comparisons for GTPs and IRs in schistosomes.

Supplementary Table 2. Genes involved in the insulin pathway in schistosomes.

Supplementary Table 3. Genes involved in glycolysis in schistosomes.
