# Supplementary figures and images for: Revisiting glucose uptake and metabolism in schistosomes: new molecular insights for improved schistosomiasis therapies
Source: Front Genet. 2014 Jun 11;5:176. doi: 10.3389/fgene.2014.00176 (PMC4052099; doi:10.3389/fgene.2014.00176)

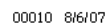

Supplement: Supplementary file 1 [file DataSheet1.ZIP › Supp Fig 1.PDF]
