# Supplementary material for: Revisiting glucose uptake and metabolism in schistosomes: new molecular insights for improved schistosomiasis therapies
Source: Front Genet. 2014 Jun 11;5:176. doi: 10.3389/fgene.2014.00176 (PMC4052099; doi:10.3389/fgene.2014.00176)
Supplement: Supplementary file 1 [file DataSheet1.ZIP › Supp Table 2.DOC]

Supplementary Table 2. Genes involved in the insulin signalling pathway in *S. japonicum, S. mansoni* and *S. haematobium*.

| **Orthology** | **Gene name inferred from electronic annotation** | **Gene ID in**  ***S. japonicum*** | **Gene ID in**  ***S. mansoni*** | **Gene ID in**  ***S. haematobium*** |
| --- | --- | --- | --- | --- |
| K07192 | flotillin | Sjp_0211790 | Smp_033970.1..pep | Sh102424 |
| K07193 | SH2B adaptor protein 2 (APS) | Sjp_0030440 | Smp_154870.1..pep | Sh101705 |
| K04707 | similar to CBL E3 ubiquitin protein ligase(Cb1) | Sjp_0077150 | Smp_005020.1..pep | Sh103248 |
| K04438 | proto-oncogene C-crk(CrkII) | Sjp_0202350 | Smp_000640.1..pep | Sh105184 |
| K06277 | Rap guanine nucleotide exchange factor 1(GRF1) | Sjp_0101370 | Smp_144600.1..pep | Sh101829 |
| K07194 | Ras homolog gene family, member Q(TC10) | Sjp_0077600 | Smp_025740.1..pep | Sh106039 |
| K07195 | exocyst complex component 7 (Exo70) | Sjp_0062940 | Smp_136580.1..pep | Sha100273 |
| K07191 | facilitated glucose tran sporter, member 4(GLUT4) | Sjp_0093040 | Smp_012440.1..pep | Sh102612 |
| K04527 | insulin receptor-1 | ACT20714  (PubMed) | AF314754  (PubMed) | -- |
|  | insulin receptor-2 | Sjp_0035100 | Smp_074030.1..pep | Sha105882 |
| K05696 | protein tyrosine phosphatase, non-receptor type 1 (PTP1B) | Sjp_0089010 | Smp_139930.1..pep | Sha105375 |
| K04440 | mitogen-activated protein kinase 10 (JNK) | Sjp_0052340 | Smp_172220.1..pep | Sha105994 |
| K00922 | phosphoinositide-3-kinase, regulatory subunit 3 (PI3K) | Sjp_0075990 | Smp_196030.1..pep | Sha105620 |
| K06276 | 3-phosphoinositide dependent protein kinase-1 (PDK1/2) | Sjp_0035230 | Smp_094250.1..pep | Sha103938 |
| K06069 | protein kinase C (aPKC) | Sjp_0054180 | Smp_096310.1..pep | Sha101385 |
| K07197 | sterol regulatory element binding factor 1(SREBP-1c) | Sjp_0091940 | Smp_000530.1..pep | Sha103631 |
| K07198 | 5'-AMP-activated protein kinase, catalytic alpha subunit (AMPK) | Sjp_0202490 | Smp_214100.1..pep | Sha103189 |
| K01961 | acetyl-Coenzyme A carboxylase beta (ACC) | Sjp_0081470 | Smp_123710.1..pep | Sha103751 |
| K00850 | Phosphofructokinase (PYK) | Sjp_0211970 | Smp_043670.1..pep | Sha101596 |
| K00873 | pyruvate kinase (GK) | Sjp_0201440 | Smp_065610.1..pep | Sha102025 |
| K03841 | fructose-1,6-bisphosphatase I (FBP) | Sjp_0215060 | Smp_097370.1..pep | --- |
| K01596 | phosphoenolpyruvate carboxykinase 2 (PEPCK) | Sjp_0202690 | Smp_005880.1..pep | Sha100954 |
| K04456 | RAC serine/threonine-protein kinase (AKt) | Sjp_0112070 | Smp_073930.1..pep | Sha100024 |
| K03083 | glycogen synthase kinase 3 beta (GSK-3β) | Sjp_0026020 | Smp_008260.1..pep | Sha104108 |
| K00693 | Glycogen (starch) synthase (GYS) | Sjp_0026410 | Smp_018260.1..pep | --- |
| K06269 | protein phosphatase 1, catalytic subunit (PP1) | Sjp_0033580 | Smp_028990.1..pep | Sha100553 |
| K00871 | phosphorylase kinase gamma subunit (PHK) | Sjp_0035630 | Smp_149040.1..pep | Sha103295 |
| K00688 | starch phosphorylase (PYG) | Sjp_0083470 | Smp_039750.1..pep | Sha104282 |
| K04345 | protein kinase A (PKA) | Sjp_0052410 | Smp_164380.1..pep | Sha100054 |
| K07188 | hormone-sensitive lipase (HSL) | Sjp_0004410 | Smp_000130.1..pep | Sha102342 |
| K07203 | FKBP12-rapamycin complex-associated protein (mTOR) | Sjp_0089220 | Smp_122910.1..pep | Sha103011 |
| K07204 | regulatory associated protein of mTOR (Raptor) | Sjp_0032410 | Smp_159000.1..pep | Sha104942 |
| K04688 | p70 ribosomal S6 kinase (p70S6K) | Sjp_0048140 | Smp_213390.1..pep | Sha100632 |
| K02991 | small subunit ribosomal protein S6e (S6) | Sjp_0074090 | Smp_053130.1..pep | Sha105919 |
| K07205 | eukaryotic translation initiation factor 4E binding protein 1 | Sjp_0023320 | Smp_076550.1..pep | Sha102317 |
| K03259 | translation initiation factor eIF-4E (eIF4E) | Sjp_0071720 | Smp_001500.1..pep | Sha103208 |
| K06279 | src homology 2 domain-containing transforming protein C (SHC) | Sjp_0041400 | Smp_196040.1..pep | Sha101705 |
| K04364 | growth factor receptor bound protein 2 (GRB2) | Sjp_0070740 | Smp_062950.1..pep | Sha102522 |
| K03099 | Son of sevenless (SOS) | Sjp_0019820 | Smp_199010.1..pep | Sha104240 |
| K07827 | GTPase Hras (Ras) | Sjp_0216000 | Smp_179910.1..pep | Sha104090 |
| K04365 | A-Raf proto-oncogene serine/threonine-protein kinase (Raf) | Sjp_0100500 | Smp_176990.1..pep | Sha101467 |
| K04368 | mitogen-activated protein kinase kinase 1(MEK1/2) | Sjp_0093560 | Smp_151670.1..pep | --- |
| K04371 | mitogen activated protein kinase 3 (ERK1/2) | Sjp_0044730 | Smp_047900.1..pep | Sha103468 |
| K04375 | ETS domain-containing protein Elk-1 (Elk1) | Sjp_0000630 | Smp_130870.1..pep | Sha100222 |
