# Supplementary material for: Revisiting glucose uptake and metabolism in schistosomes: new molecular insights for improved schistosomiasis therapies
Source: Front Genet. 2014 Jun 11;5:176. doi: 10.3389/fgene.2014.00176 (PMC4052099; doi:10.3389/fgene.2014.00176)
Supplement: Supplementary file 1 [file DataSheet1.ZIP › Supp Table 3.DOC]

Supplementary Table 3. Genes involved in glycolysis in *S. mansoni*, *S. japonicum* and *S.haematobium*.

| **Orthology** | **Gene name inferred from electronic annotation annotation** | **Gene ID in**  ***S. japonicum*** | **Gene ID in**  ***S. mansoni*** | **Gene ID in**  ***S. haematobium*** |
| --- | --- | --- | --- | --- |
| K01835 | phosphoglucomutase 1 | [Sjp_0073670](http://chgc.sh.cn/japonicum/Sjpathway/blast/K01835.html#Sjp_0073670) | Smp_180530.4..pep | Sha103579 |
| K01810 | glucose phosphate isomerise | [Sjp_0301250](http://chgc.sh.cn/japonicum/Sjpathway/blast/K01810.html#Sjp_0301250) | Smp_022400.1..pep | Sha105820 |
| K03841 | fructose-1,6-bisphosphatase I | Sjp_0215060 | Smp_097370.1..pep | --- |
| K00850 | 6-phosphofructokinase | Sjp_0211970 | Smp_043670.1..pep | Sha101596 |
| K01622 | fructose-bisphosphate aldolase | Sjp_0031630 | Smp_042160.2..pep | --- |
| K01810 | glucose-6-phosphate isomerise | Sjp_0301250 | Smp_022400.1..pep | Sha105820 |
| K00844 | hexokinase 2 | Sjp_0302060 | Smp_043030.1..pep | Sha104778 |
| K01803 | triosephosphate isomerase (TIM) | Sjp_0304690 | Smp_003990.1..pep | --- |
| K00134 | glyceraldehyde 3-phosphate dehydrogenase | Sjp_0025800 | Smp_056970.1..pep | Sha105909 |
| K00927 | phosphoglycerate kinase 2 | Sjp_0031010 | Smp_214060.1..pep | Sha103905 |
| K01512 | acylphosphatase 2, muscle type | Sjp_0098030 | Smp_154480.1..pep | --- |
| K01834 | phosphoglycerate mutase | Sjp_0304750 | Smp_096760.1..pep | Sha105217 |
| K01689 | enolase | Sjp_0209670 | Smp_024110.1..pep | Sha101927 |
| K00873 | pyruvate kinase | Sjp_0201440 | Smp_065610.1..pep | Sha102025 |
| K00016 | lactate dehydrogenase A | Sjp_0110090 | Smp_038950.1..pep | Sha102033 |
| K00160 | pyruvate dehydrogenase | Sjp_0206920 | Smp_002510.1..pep | Sha100181 |
| K00627 | pyruvate dehydrogenase E2 component | Sjp_0216460 | Smp_212190.1..pep | Sha101645 |
| K00382 | dihydrolipoamide dehydrogenase | Sjp_0205700 | Smp_046740.1..pep | Sha102231 |
| K00001 | alcohol dehydrogenase; | Sjp_0313140 | Smp_044440.1..pep | Sha105634 |
| K00002 | alcohol dehydrogenase | Sjp_0217190 | Smp_053220.1..pep | Sha101024 |
| K00128 | aldehyde dehydrogenase | Sjp_0208240 | Smp_050390.1..pep | Sha100859 |
| K00129 | aldehyde dehydrogenase | Sjp_0057270 | Smp_172730.1..pep | Sha101062 |
| K01895 | acetyl-CoA synthetase | Sjp_0212080 | Smp_168520.1..pep | Sha105795 |
