# Supplementary material for: Revisiting glucose uptake and metabolism in schistosomes: new molecular insights for improved schistosomiasis therapies
Source: Front Genet. 2014 Jun 11;5:176. doi: 10.3389/fgene.2014.00176 (PMC4052099; doi:10.3389/fgene.2014.00176)
Supplement: Supplementary file 1 [file DataSheet1.ZIP › Supp Table 1.DOC]

| **Schistosome species** | **GTP-1** | | **GTP-4** | | **IR-1** | | | **IR-2** | | |
| --- | --- | --- | --- | --- | --- | --- | --- | --- | --- | --- |
|  | Genebank ID | Homology with *S. mansoni* sequence | Genebank ID | Homology with *S. mansoni* sequence | Genebank ID | Homology with *S. mansoni* sequence | Homology with HIR sequence | Genebank ID | Homology with *S. mansoni* sequence | Homology with HIR sequence |
| ***S. mansoni*** | AAA19731 (PubMed) | --- | AAA19733  (PubMed) | --- | AF314754  (PubMed) | --- | 24.9% | AAV65745  (PubMed) | --- | 26.1% |
| ***S. japonicum*** | Sjp_0093040  (GeneDB) | 84% | AAW24832 *(PubMed) and Sjc_0219380*  (chgc.sh.cn/japonicum/Resource.html) | --- | ACT20714  (PubMed) | 69.7% | 24.8% | ACT20715  (PubMed) | 72.9% | 25.2% |
| ***S. haematobium*** | Sha102612  (GeneDB) | 87.9% | Sha102063  (GeneDB) | 89.9% | --- | --- | --- | Sha105882  (GeneDB) | 89.8% | 25.7% |

Supplementary Table 1. Genebank ID and amino acid sequence homology comparisons for glucose transport protein (GTP)-1, GTP-4 and insulin receptor (IR)-1 and IR-2 in *S. mansoni, S. japonicum* and *S. haematobium*

Note: * partial sequence; HIR, human insulin receptor
